# Integrated Multi-Omics Analysis Reveals Activation of the PPAR Signaling Pathway by Koumiss in Experimental Ulcerative Colitis

**DOI:** 10.3390/ijms27093821

**Published:** 2026-04-25

**Authors:** Guanglin Guo, Pinjie Bao, Bolag Altan, Bateer Siqin

**Affiliations:** 1 College of Mongolian Medicine and Pharmacy, Inner Mongolia Medical University, Hohhot 010110, China; gglgjmy@163.com; 2 Inner Mongolia Traditional Chinese and Mongolian Medical Research Institute, Hohhot 010010, China; baopinjie2019@163.com

**Keywords:** koumiss, ulcerative colitis, PPAR signaling, proteomics, network pharmacology, functional food, multi-omics

## Abstract

Ulcerative colitis (UC) is a chronic inflammatory bowel disease characterized by persistent mucosal inflammation and dysregulated immune–metabolic responses. Koumiss, a traditional fermented mare’s milk, has long been used in ethnomedicine for gastrointestinal disorders; however, its molecular mechanisms in UC remain unclear. In this study, an integrated multi-omics approach combining network pharmacology, quantitative proteomics, and molecular docking was employed to elucidate the therapeutic mechanism of koumiss powder (KP) in a dextran sulfate sodium (DSS)-induced murine colitis model. Network pharmacology identified twelve bioactive compounds targeting fourteen UC-associated proteins, predominantly enriched in the peroxisome proliferator-activated receptor (PPAR) signaling pathway. In vivo experiments demonstrated that high-dose KP significantly alleviated disease activity, improved colon shortening and histopathological injury, reduced serum TNF-α and IL-6 levels, and restored anti-inflammatory cytokines IL-4 and IL-10. Proteomic analysis further revealed activation of the PPAR signaling pathway, with significant upregulation of Plin4 and Sorbs1. Immunofluorescence staining further confirmed that KP restored the expression of PPARA and increased the levels of Plin4 and Sorbs1 in colonic tissues. Molecular docking confirmed strong binding affinities between key koumiss-derived lipid metabolites, including 13(S)-HOTrE and stearoyl ethanolamide, and PPAR-related target proteins. Collectively, these findings demonstrate that koumiss exerts protective effects against experimental UC primarily through activation of PPAR-mediated lipid metabolic and anti-inflammatory pathways. This study provides mechanistic insight into the biological activity of koumiss and highlights the value of multi-omics integration in natural product research.

## 1. Introduction

Ulcerative colitis (UC) is a chronic, relapsing inflammatory bowel disease characterized by persistent mucosal inflammation of the colon and rectum [1,2,3]. The global prevalence of UC has exceeded over five million cases and continues to rise steadily, posing substantial clinical and socioeconomic burdens [4]. Although conventional treatments, including aminosalicylates, corticosteroids, and immunosuppressants, can alleviate symptoms, many patients experience incomplete remission, frequent relapse, adverse effects, and high long-term treatment costs [5,6,7]. Therefore, safer and more effective complementary or alternative therapeutic strategies are urgently needed [8].

Natural products and functional foods have attracted increasing attention for their potential to regulate intestinal inflammation and restore mucosal homeostasis with lower toxicity [9]. Koumiss, a fermented beverage produced by the co-fermentation of mare’s milk with lactic acid bacteria and yeast, is traditionally consumed in East Asian nomadic regions as both a nutritional supplement and a medicinal food [10,11,12]. Koumiss contains diverse bioactive constituents, including short-chain fatty acids, peptides, amino acids, vitamins, and microbial metabolites, which collectively exhibit anti-inflammatory, antimicrobial, immunomodulatory, and gastrointestinal barrier-protective properties [13,14]. Recent studies further suggest that koumiss may regulate host lipid metabolism, immune responses, and gut microbial homeostasis, implying potential therapeutic value in chronic inflammatory diseases [15,16,17,18,19]. In parallel, increasing evidence indicates that peroxisome proliferator-activated receptors (PPARs), especially PPARA, are central regulators of intestinal immunometabolism, epithelial barrier integrity, and inflammatory resolution in UC [20,21,22,23]. Pharmacological or nutritional activation of PPAR-related signaling has been reported to ameliorate DSS-induced colitis and other inflammation-associated intestinal disorders [24,25].

Despite accumulating evidence suggesting the beneficial effects of koumiss in gastrointestinal disorders, the specific bioactive compounds, molecular targets, and regulatory pathways underlying its protective role in ulcerative colitis remain largely undefined. In particular, it is still unclear whether koumiss exerts its anti-colitic effects through coordinated modulation of lipid metabolic signaling pathways and whether PPAR-related downstream effectors are involved in this process. In addition, because koumiss is a fermented dairy product, its beneficial effects may also involve microbiota-dependent mechanisms, which should be considered when interpreting its activity [15].

We hypothesized that koumiss-derived lipid metabolites ameliorate intestinal inflammation through modulation of the peroxisome proliferator-activated receptor (PPAR) signaling pathway [16]. To test this hypothesis, we employed an integrated multi-omics framework combining network pharmacology, quantitative proteomics, molecular docking analysis, and in vivo validation using a dextran sulfate sodium (DSS)-induced murine colitis model [17]. In the following study, we further supplemented the mechanistic analysis by examining the expression changes of PPARA and its downstream PPAR-related proteins Plin4 and Sorbs1 in colonic tissues. This systematic approach aimed to elucidate the molecular mechanism underlying the therapeutic effects of koumiss and to clarify its potential role in immunometabolic regulation in ulcerative colitis.

## 2. Results

### 2.1. Network Pharmacological Analysis of Koumiss Bioactive Components

**Figure 1:**
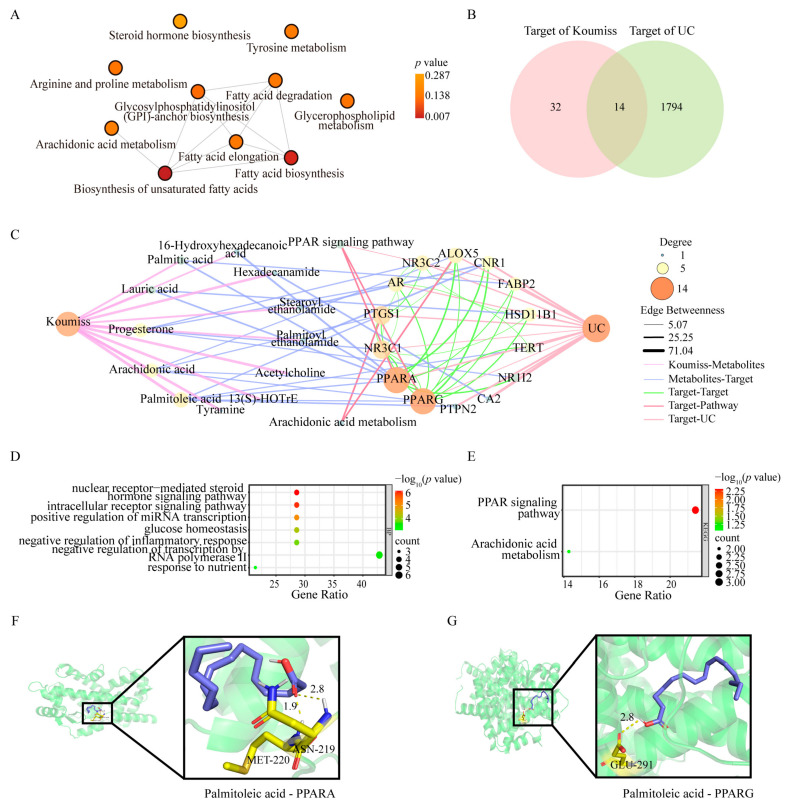
Network pharmacological analysis of koumiss-derived bioactive compounds in ulcerative colitis. (**A**) Pathway enrichment analysis of candidate active compounds identified from koumiss, showing the major lipid metabolism-related pathways potentially involved. (**B**) Venn diagram showing the intersection between ulcerative colitis (UC)-associated targets and predicted targets of koumiss-derived active compounds. (**C**) Integrated compound–target network and protein–protein interaction (PPI) network of the overlapping targets; hexagons represent active compounds, circles represent shared targets, and node size reflects degree centrality. (**D**) Gene Ontology (GO) biological process enrichment analysis of the 14 intersected targets. (**E**) Kyoto Encyclopedia of Genes and Genomes (KEGG) pathway enrichment analysis of the intersected targets, highlighting the PPAR signaling pathway and arachidonic acid metabolism. (**F**,**G**) Representative molecular docking conformations of palmitoleic acid with PPARA (**F**) and PPARG (**G**). Residues involved in the interactions are shown as sticks, and hydrogen bonds are indicated by yellow dashed lines.

Our previous LC–MS analysis identified 66 chemical constituents in koumiss [17]. Among them, 2 compounds were screened through the TCMSP database, while 20 compounds met the screening criteria in both the SwissADME and pkCSM databases. After integration and deduplication, 22 active compounds were retained as candidate bioactive ingredients (Table 1). These compounds were primarily fatty acids and lipid mediators, indicating a potential role in metabolic and inflammatory regulation. Functional enrichment analysis revealed that these candidate compounds were mainly associated with lipid metabolic processes. As illustrated in Figure 1A, significantly enriched pathways included fatty acid biosynthesis, fatty acid elongation, arachidonic acid metabolism, glycerophospholipid metabolism, and steroid hormone biosynthesis, suggesting that lipid metabolic reprogramming may represent a principal mechanistic axis of koumiss activity. Venn intersection analysis identified 14 overlapping targets between koumiss-predicted targets and UC-associated genes (Figure 1B). Protein–protein interaction (PPI) network construction demonstrated strong connectivity among these targets (Figure 1C), highlighting a tightly interconnected regulatory module. Notably, individual bioactive compounds were predicted to interact with multiple targets, while several UC-related targets were simultaneously modulated by multiple koumiss-derived metabolites, reflecting a typical multi-component–multi-target pharmacological pattern. Gene Ontology enrichment analysis indicated that these intersecting targets were significantly involved in inflammatory response regulation and nuclear receptor-mediated signaling (Figure 1D). KEGG pathway analysis further demonstrated significant enrichment in the PPAR signaling pathway and arachidonic acid metabolism (Figure 1E), both of which are critically implicated in intestinal inflammation and mucosal homeostasis.

To further validate these predicted interactions, molecular docking analysis was performed between major koumiss-derived compounds and core PPAR-related proteins. As shown in Table 2, most active ingredients exhibited favorable binding energies (<−5.0 kcal/mol), indicating stable ligand–receptor interactions. Among them, palmitoleic acid demonstrated relatively strong binding affinity (<−6.0 kcal/mol) toward both PPARA and PPARG. Representative docking conformations are shown in Figure 1F,G, revealing hydrogen bonding and hydrophobic interactions within the ligand-binding domains.

Collectively, these results suggest that koumiss exerts its anti-ulcerative colitis effects primarily through coordinated regulation of the PPAR signaling pathway and lipid metabolic networks, particularly arachidonic acid-related pathways.

### 2.2. Alleviation Effects of KP in DSS-Induced UC Mice

**Figure 2:**
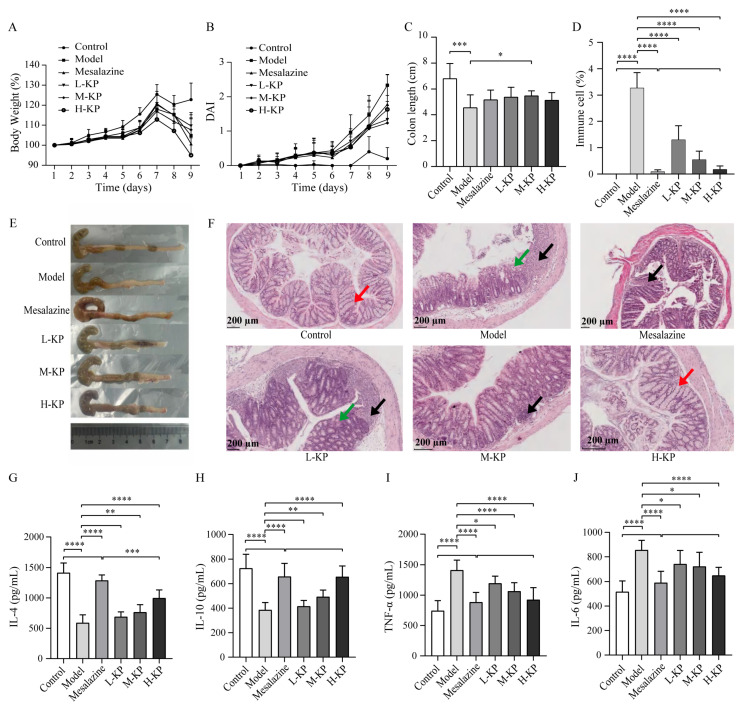
Protective effects of koumiss powder on DSS-induced ulcerative colitis in mice. (**A**) Changes in body weight during the experimental period. (**B**) Disease Activity Index (DAI) scores evaluating clinical severity. (**C**) Colon length measurements reflecting the degree of colonic inflammation (n = 10/group). (**D**) Quantification of inflammatory cell infiltration in five representative microscopic fields of colonic tissues (n = 5/group). (**E**) Representative macroscopic images of colons from each experimental group. (**F**) Representative hematoxylin and eosin (H&E)-stained colon sections showing histopathological alterations. Black arrows indicate inflammatory cell infiltration; red arrows indicate goblet cells; green arrows indicate crypt structure damage. Scale bar = 200 μm. (**G**–**J**) Serum cytokine levels, including the anti-inflammatory cytokines IL-4 (**G**) and IL-10 (**H**), and the pro-inflammatory cytokines TNF-α (**I**) and IL-6 (**J**) (n = 10/group). Data are presented as mean ± SEM. * *p* < 0.05, ** *p* < 0.01, *** *p* < 0.001, **** *p* < 0.0001. KP, koumiss powder; L-KP, low-dose KP group; M-KP, medium-dose KP group; H-KP, high-dose KP group.

To evaluate the therapeutic efficacy of KP in DSS-induced UC, clinical parameters, histopathological alterations, and systemic inflammatory responses were systematically assessed. As shown in Figure 2A,B, DSS administration resulted in a progressive increase in the disease activity index (DAI) and significant body weight loss compared with the control group, indicating successful establishment of the acute UC model. KP intervention markedly attenuated these clinical symptoms, as evidenced by reduced DAI scores and mitigation of DSS-induced weight loss. Notably, the M-KP and H-KP groups exhibited more pronounced protective effects. Colon length, a macroscopic indicator of colonic inflammation severity, was significantly shortened in the model group. KP treatment, particularly at medium dosage (M-KP), significantly alleviated DSS-induced colon shortening (Figure 2C, n = 10/group). Consistently, immune cell infiltration was markedly increased in the model group, whereas KP administration effectively reduced inflammatory cell accumulation (Figure 2D, n = 5/group).

Gross morphological observation further supported these findings (Figure 2E). Compared with the thin and soft colonic tissue observed in the control group, DSS-treated mice exhibited rigid, thickened colons with evident edema and hyperplasia. KP treatment alleviated these pathological changes to varying degrees. Histopathological examination by H&E staining revealed severe mucosal injury in the model group, characterized by epithelial disruption, crypt destruction, goblet cell depletion, inflammatory cell infiltration, and muscular layer edema (Figure 2F). In contrast, both mesalazine and KP-treated groups showed substantial histological improvement. Importantly, the H-KP group demonstrated better preservation of goblet cells, relatively intact crypt architecture, a more continuous mucus layer, and only mild inflammatory infiltration, suggesting enhanced mucosal barrier protection.

To further determine whether KP-mediated histological recovery was associated with modulation of systemic inflammation, serum cytokine levels were quantified by ELISA (n = 10). DSS exposure significantly suppressed anti-inflammatory cytokines IL-4 and IL-10 (Figure 2G,H) while markedly elevating pro-inflammatory cytokines TNF-α and IL-6 (Figure 2I,J) compared with the control group (all *p* < 0.0001). KP treatment effectively reversed this imbalance by restoring IL-4 and IL-10 levels and reducing TNF-α and IL-6 production. Notably, the H-KP group exhibited comparable efficacy to mesalazine, with no significant differences observed in IL-10, TNF-α, or IL-6 levels between the two groups.

Collectively, these results demonstrate that KP effectively alleviates DSS-induced acute UC by improving clinical symptoms, preserving intestinal morphology and mucosal architecture, and restoring systemic inflammatory homeostasis. The therapeutic efficacy of high-dose KP is comparable to that of mesalazine, highlighting its potential as a promising candidate for UC management.

### 2.3. H-KP Remodels the Colonic Proteome and Activates the PPAR Signaling Pathway

To investigate the molecular basis of KP-mediated protection, label-free LC–MS/MS proteomic analysis was performed on colon tissues. A total of 7241 to 33,547 peptides were identified across samples and 1096–4162 proteins were identified across samples (FDR < 0.01), with most proteins distributed between 10–60 kDa.

**Figure 3:**
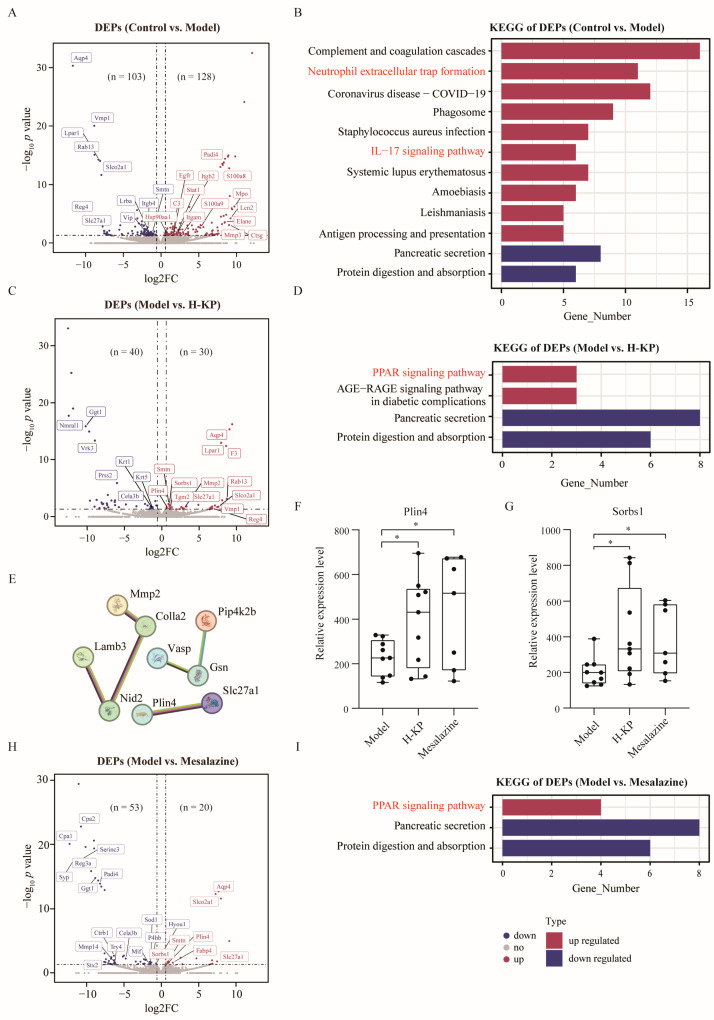
Proteomic profiling and pathway analysis of koumiss treatment in DSS-induced ulcerative colitis mice. (**A**) Volcano plot showing differentially expressed proteins (DEPs) between the model and control groups (FDR-adjusted *p* < 0.05). (**B**) KEGG pathway enrichment analysis of DEPs identified in the model group relative to the control group. (**C**) Volcano plot of DEPs between the H-KP and model groups. (**D**) KEGG pathway enrichment analysis of DEPs in the H-KP group compared with the model group. (**E**) Protein–protein interaction (PPI) network of DEPs regulated by H-KP treatment, highlighting hub proteins within the PPAR-related regulatory network. (**F**,**G**) Relative expression levels of Plin4 (**F**) and Sorbs1 (**G**) identified by proteomic analysis in colonic tissues. (**H**) Volcano plot of DEPs between the mesalazine and model groups. (**I**) KEGG pathway enrichment analysis of DEPs in the mesalazine group compared with the model group. In box plots, the center line represents the median, box limits represent the upper and lower quartiles, and whiskers represent the range. * *p* < 0.05.

Compared with the control group, DSS treatment resulted in 128 up-regulated and 103 down-regulated proteins (Figure 3A). Enrichment analysis showed that up-regulated proteins were mainly associated with proteolysis and coagulation processes, whereas down-regulated proteins were involved in anion transport (Supplementary Figure S1A). KEGG analysis revealed significant enrichment of IL-17 signaling and neutrophil extracellular trap formation in the model group (Figure 3B).

H-KP treatment led to 30 up-regulated and 40 down-regulated proteins compared with the model group (Figure 3C). These DEPs were enriched in actin filament dynamics, protein polymerization, keratinocyte differentiation, and amino acid metabolism (Supplementary Figure S1B). KEGG analysis revealed activation of the PPAR signaling pathway, concurrent with inhibition of pancreatic secretion and protein digestion/absorption pathways (Figure 3D). PPI analysis identified Plin4 and Sorbs1 as hub proteins within the PPAR-related regulatory network (Figure 3E). These two proteins were highlighted because they were significantly regulated in the proteomic dataset, enriched in the PPAR signaling pathway, and positioned centrally in the protein–protein interaction network, indicating potential biological relevance to lipid metabolic regulation and inflammatory homeostasis. The PPAR-associated proteins Plin4 (O88492) and Sorbs1 (Q62417) (Figure 3F,G, both *p* < 0.05), which showed expression trends consistent with the therapeutic effects of H-KP, were significantly upregulated in the H-KP and mesalazine groups compared with the model group.

Mesalazine administration resulted in 20 up-regulated and 53 down-regulated proteins compared to the model group (Figure 3H). These DEPs were associated with fatty acid transport, cellular responses to metal ions, regulation of apoptosis, and skeletal muscle acetylcholine-gated channel clustering (Supplementary Figure S1C). Notably, similar KEGG enrichment patterns, including activation of the PPAR signaling pathway (Figure 3I). In contrast, L-KP (Supplementary Figure S2A) and M-KP (Supplementary Figure S2B) group demonstrated relatively fewer regulated proteins involved in actin cytoskeleton organization (Supplementary Figure S2C) and fatty acid/biotin transport (Supplementary Figure S2D). However, neither L-KP nor M-KP significantly influenced UC-associated pathways (Supplementary Figure S2E).

These findings reveal that H-KP exerts therapeutic effects in DSS-induced UC through regulation of the PPAR signaling pathway, with Plin4 and Sorbs1 identified as representative downstream proteins associated with this response.

### 2.4. Overlapping and Reversal Patterns of DEPs Between H-KP and Mesalazine

**Figure 4:**
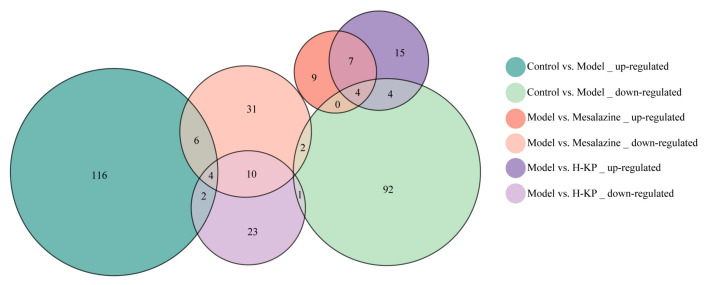
Comparative overlap and reversal analysis of differentially expressed proteins across treatment groups. Venn diagrams showing the overlap of upregulated and downregulated DEPs among the model vs. control, H-KP vs. model, and mesalazine vs. model comparisons. Numerical values indicate the numbers of unique and shared proteins among the different comparisons. This analysis illustrates both the common regulatory effects shared by H-KP and mesalazine and the reversal of DSS-induced proteomic alterations following treatment. vs., versus.

To compare the regulatory patterns of H-KP and mesalazine, Venn analysis was performed to identify overlapping and unique differentially expressed proteins (DEPs) among treatment groups (Figure 4). Both H-KP and mesalazine modulated a subset of common proteins. Among these shared DEPs, 11 proteins were consistently up-regulated (55% and 37% of the total up-regulated proteins in the mesalazine and H-KP groups, respectively), while 14 proteins were commonly down-regulated (26% and 35%, respectively). These findings indicate partial overlap in the molecular responses induced by the two treatments.

Importantly, H-KP exhibited a pronounced reversal effect on DSS-induced proteomic alterations. Specifically, 8 proteins (27%) that were down-regulated in the model group were restored by H-KP treatment, a greater number than observed in the mesalazine group. In addition, H-KP down-regulated 6 proteins (13%) that had been up-regulated by DSS modeling. By comparison, mesalazine down-regulated 10 proteins (19%) that were elevated in the model group.

Overall, while H-KP and mesalazine share partially overlapping regulatory profiles, H-KP demonstrated a stronger trend toward reversing UC-associated proteomic disturbances. These results suggest that H-KP exerts therapeutic effects through both shared and distinct molecular mechanisms.

### 2.5. Molecular Docking Analysis Predicts Strong Interactions Between Koumiss-Derived Metabolites and PPAR-Related Targets

**Figure 5:**
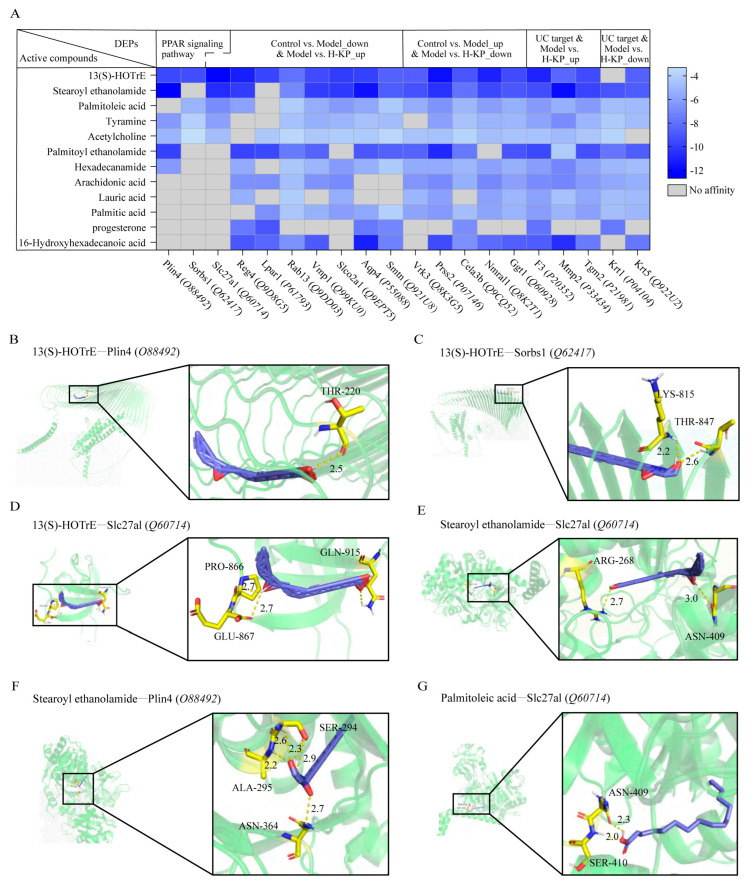
Molecular docking analysis of koumiss-derived bioactive compounds with PPAR-related targets and ulcerative colitis-associated proteins. (**A**) Heatmap of AutoDock Vina binding energies (BE) between 12 koumiss-derived active compounds and representative proteins associated with UC and the PPAR-related regulatory network. The color gradient indicates predicted binding affinity (blue, stronger; light blue, weaker; gray, no predicted affinity), and values <−5 kcal/mol were considered indicative of favorable binding. (**B**–**G**) Representative docking conformations of key compound–target pairs, including 13(S)-HOTrE−Plin4 (**B**), 13(S)-HOTrE−Sorbs1 (**C**), 13(S)-HOTrE−Slc27a1 (**D**), stearoyl ethanolamide−Slc27a1 (**E**), stearoyl ethanolamide−Plin4 (**F**), and palmitoleic acid−Slc27a1 (**G**), with the corresponding binding energies shown in each panel.

Molecular docking analysis was performed to evaluate the binding affinity between the 12 identified active compounds and DEPs exhibiting reversed expression trends after H-KP treatment. The heatmap illustrates the overall binding energy distribution between koumiss-derived metabolites and UC-associated targets (Figure 5A), indicating widespread and stable ligand–receptor interactions.

Among the active ingredients, 13(S)-HOTrE exhibited the strongest and most consistent binding affinities toward key proteins involved in the PPAR signaling pathway, including Plin4 (*O88492*) (Figure 5B; −9.6 kcal/mol), Sorbs1 (*Q62417*) (Figure 5C; −9.4 kcal/mol), and Slc27a1 (*Q60714*) (Figure 5D; −12.2 kcal/mol). These interactions were stabilized by multiple hydrogen bonds and hydrophobic contacts, suggesting high binding stability.

Similarly, stearoyl ethanolamide exhibited strong affinity for Slc27a1(*Q60714*) (Figure 5E; −10.7 kcal/mol) and Plin4 (*O88492*) (Figure 5F; −12.7 kcal/mol), further supporting lipid-mediated regulation of PPAR-related targets. In addition, palmitoleic acid revealed a favorable binding affinity with Slc27a1 (*Q60714*) (Figure 5G; −6.8 kcal/mol).

Collectively, these docking results suggest that fatty acid-derived metabolites in koumiss may interact with proteins associated with the PPAR-related regulatory network. Together with the proteomic and immunofluorescence results, these findings support the possibility that H-KP exerts therapeutic effects in UC through modulation of PPARα-associated lipid metabolic and anti-inflammatory pathways. However, the docking results should be interpreted as supportive structural evidence rather than direct proof of target engagement.

### 2.6. KP Restores PPARA, Plin4, and Sorbs1 Expression in Colonic Tissues

**Figure 6:**
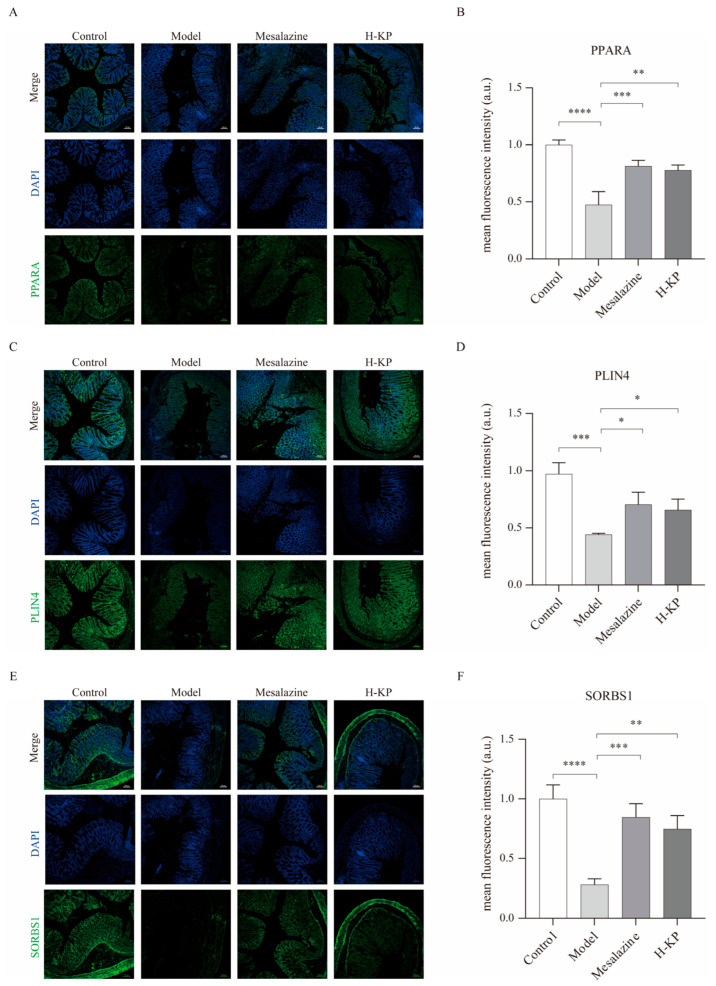
Immunofluorescence validation of PPARA, Plin4, and Sorbs1 expression in colonic tissues. Representative immunofluorescence images showing the expression of PPARA (**A**), Plin4 (**C**), and Sorbs1 (**E**) in colon tissues from the Control, Model, Mesalazine, and H-KP groups. Corresponding quantitative analysis of fluorescence intensity is shown for PPARA (**B**), Plin4 (**D**), and Sorbs1 (**F**). Nuclei were counterstained with DAPI (blue), and positive staining is shown in green. DSS treatment markedly reduced the expression of PPARA, Plin4, and Sorbs1, whereas mesalazine and H-KP treatment restored their expression levels. Data are presented as mean ± SEM. * *p* < 0.05, ** *p* < 0.01, *** *p* < 0.001, **** *p* < 0.0001. H-KP, high-dose koumiss powder. Scale bar = 100 μm.

To further validate the involvement of the PPAR signaling pathway in the protective effects of koumiss, immunofluorescence staining was performed to assess the expression of PPARA, Plin4, and Sorbs1 in colonic tissues (Figure 6). As shown in Figure 6A,B, PPARA fluorescence intensity was markedly decreased in the DSS model group compared with the control group, indicating suppression of PPARA-related signaling under inflammatory conditions. Both mesalazine and high-dose koumiss powder (H-KP) treatment significantly restored PPARA expression relative to the model group.

Consistent with this finding, the expression levels of the downstream PPAR-related proteins Plin4 and Sorbs1 were also markedly reduced in the model group, whereas both mesalazine and H-KP treatment significantly increased their fluorescence intensities (Figure 6C–F). Specifically, Plin4 expression was significantly elevated after both mesalazine and H-KP intervention compared with the model group, and a similar restoration trend was observed for Sorbs1.

Overall, these results provide direct tissue-level evidence that KP reverses DSS-induced downregulation of PPARA, Plin4, and Sorbs1 in the colon, further supporting the conclusion that koumiss ameliorates experimental ulcerative colitis, at least in part, through activation of the PPARA-associated signaling axis.

## 3. Discussion

Koumiss has traditionally been used for gastrointestinal disorders; however, its molecular mechanism in ulcerative colitis (UC) has not been systematically clarified [18,19]. In the present study, we integrated network pharmacology, in vivo validation, quantitative proteomics, and molecular docking analyses to investigate the mechanistic basis of koumiss in DSS-induced experimental colitis. The combined results indicate that koumiss exerts anti-colitic effects through coordinated regulation of inflammatory responses and lipid metabolic pathways, with PPAR-related signaling representing a key mechanistic feature [20].

Network pharmacology analysis suggested that lipid-related pathways, particularly the PPAR signaling pathway and arachidonic acid metabolism, represent central regulatory axes of koumiss bioactivity [21,22]. PPARs are well-established regulators of lipid homeostasis, epithelial barrier integrity, and inflammatory responses in the intestine [23,24]. Dysregulation of PPAR signaling has been implicated in UC pathogenesis, and pharmacological activation of PPAR-γ has demonstrated protective effects in experimental colitis models [25]. Our findings that multiple koumiss-derived fatty acids and lipid mediators were predicted to target PPAR-associated targets supports the notion that lipid-sensitive regulatory mechanisms contribute importantly to the anti-inflammatory effects of koumiss. Given the close interplay between mucosal inflammation and disturbed lipid metabolism in UC, the enrichment of PPAR-related pathways provides a biologically coherent framework for interpreting the therapeutic effects observed in this study [26].

The mechanistic predictions were corroborated by in vivo experiments. High-dose koumiss powder significantly alleviated disease activity, improved histopathological injury, and restored inflammatory cytokine balance in DSS-treated mice [27]. Proteomic profiling further revealed enrichment of the PPAR signaling pathway and upregulation of Plin4 and Sorbs1, two proteins involved in lipid droplet dynamics and metabolic regulation. These proteins were selected for further interpretation because they fulfilled several criteria simultaneously: they were significantly regulated in the proteomic analysis, enriched within the PPAR signaling pathway, showed expression trends consistent with the therapeutic effect of H-KP, and occupied important positions in the protein–protein interaction network [28]. These findings indicate that koumiss may modulate immunometabolic pathways associated with intestinal inflammation [29,30]. The recovery of these proteins suggests that koumiss may alleviate colitis, at least in part, by normalizing inflammation-associated disturbances in lipid handling and intracellular metabolic signaling [12]. Such regulation may contribute not only to suppression of inflammatory injury but also to restoration of mucosal homeostasis [31].

Comparative analysis showed partial overlap between koumiss and mesalazine in regulating PPAR-related pathways, while koumiss demonstrated a broader reversal of DSS-induced proteomic alterations [32,33]. This suggests that, in addition to shared anti-inflammatory mechanisms, koumiss may exert broader multi-target regulatory effects through coordinated modulation of lipid metabolism and inflammatory signaling networks [34]. This broader regulatory profile is consistent with the compositional complexity of koumiss, which contains multiple bioactive constituents that may act in a complementary or synergistic manner.

Molecular docking provided structural support for these observations. Key lipid metabolites, including 13(S)-HOTrE and stearoyl ethanolamide, exhibited stable binding affinities toward PPAR-associated targets such as Plin4, Sorbs1, and Slc27a1 [35,36,37]. However, these results should be interpreted with caution. Docking analysis predicts potential ligand–target interactions but does not establish direct biological activity in vivo. Accordingly, the docking data in the present study should be regarded as supportive evidence that is consistent with the proteomic and pathway-level findings, rather than as definitive mechanistic proof.

Importantly, because koumiss is a fermented dairy product, its beneficial effects may also involve modulation of the gut microbiota [13]. This possibility deserves consideration when interpreting the present findings. Accumulating evidence indicates that fermented foods and microbially derived metabolites can influence intestinal inflammation by reshaping microbial communities, enhancing epithelial barrier function, and regulating mucosal immune responses [38,39]. Therefore, the protective effects of koumiss observed here may not be solely attributable to direct host metabolic regulation, but may also involve microbiota-mediated mechanisms that contribute to intestinal homeostasis.

This possibility is particularly relevant in the context of PPAR signaling. Host–microbiota interactions are increasingly recognized as important modulators of intestinal PPAR activity, and microbially derived lipid-related metabolites have been reported to influence epithelial metabolism, immune balance, and barrier integrity through PPAR-associated pathways [40]. From this perspective, the PPAR-centered effects identified in our study may reflect not only host-targeted actions of koumiss-derived compounds, but also an integrated response involving microbial metabolism and host immunometabolic regulation.

Several limitations should be acknowledged. Although the present results support the involvement of PPAR-related signaling, additional functional studies, such as pathway inhibition, reporter assays, or genetic perturbation approaches, would be required to establish causality more directly [41,42]. In addition, microbiota profiling was not performed in this study, and this absence represents an important limitation given the fermented nature of koumiss. Future studies integrating microbiome, metabolome, and host signaling analyses will be important for clarifying whether koumiss acts predominantly through direct host-target interactions, microbiota-dependent mechanisms, or the interplay between both processes [43].

Overall, this study provides multi-layered evidence that koumiss ameliorates experimental colitis primarily through modulation of PPAR-dependent lipid metabolic and anti-inflammatory pathways. At the same time, our findings support a broader mechanistic perspective in which host immunometabolic regulation and potential host–microbiota crosstalk may jointly contribute to the therapeutic effects of koumiss. This integrative view may be valuable for understanding the biological actions of fermented functional foods in UC and related inflammatory intestinal disorders [44].

## 4. Materials and Methods

### 4.1. Network Pharmacology Analysis

#### 4.1.1. Identification of Bioactive Constituents and Target Prediction

Based on our previous chemical profiling of koumiss using liquid chromatography–tandem mass spectrometry (LC-MS) [45], candidate bioactive compounds were screened using the Traditional Chinese Medicine Systems Pharmacology database (TCMSP) (retrieved 28 March 2023, from https://www.tcmsp-e.com/load_intro.php?id=43), SwissADME (retrieved 28 March 2023, from http://www.swissadme.ch/), and pkCSM platforms (retrieved 28 March 2023, from http://biosig.unimelb.edu.au/pkcsm/) [46]. To ensure favorable pharmacokinetic properties, the following criteria were applied: oral bioavailability ≥30%, drug-likeness ≥0.18, high gastrointestinal absorption, fulfillment of at least three of five drug-likeness rules, and Caco-2 permeability ≥0.9. Canonical SMILES structures of the potential active ingredients were retrieved from the PubChem database (https://pubchem.ncbi.nlm.nih.gov/, accessed on 21 April 2026) and uploaded to the SwissTargetPrediction database (http://swisstargetprediction.ch/, accessed on 21 April 2026) to predict potential targets. The screening was performed under the conditions of “*Homo sapiens*” and a probability threshold >0.2.

#### 4.1.2. Identification of UC-Associated Targets

Ulcerative colitis–related genes were collected from GeneCards, DisGeNET, OMIM, Therapeutic Target Database, and DrugBank. The GeneCards database (version 5.24) was queried with a relevance score cutoff set at the median value of 2.92, while the DisGeNET database (version 24) was screened using a gene–disease association score threshold ≥0.2, specific to UC. Additional targets were retrieved from the Online Mendelian Inheritance in Man (OMIM) database, Therapeutic Target Database (TTD, updated 10 January 2024), and DrugBank database (version 5.1.13). Following data collection, all identified targets were consolidated and duplicate entries were removed to generate a refined set of UC-associated genes.

After deduplication, intersecting genes between koumiss-derived bioactive ingredients and the curated UC-related gene set were identified. Protein–protein interaction (PPI) network analysis was performed using the STRING database (https://string-db.org/, accessed on 21 April 2026) with the search parameters restricted to Homo sapiens and a minimum interaction confidence score of 0.4. The PPI network data was imported into Cytoscape software (version 3.7.0) for visualization and further analysis. Network topology and key nodal proteins were characterized using the CentiScaPe plugin, which enabled quantitative assessment of node centrality metrics.

A compound–target–pathway integrated network was subsequently constructed to elucidate the multi-target pharmacological mechanism of koumiss.

### 4.2. Preparation of Koumiss Freeze-Dried Powder

Koumiss (from Mengma Dairy, Xilinguole, Inner Mongolia of China) was thoroughly mixed with anhydrous ethanol at a 1:1 (*v*/*v*) ratio. The mixture was then centrifuged at 4000 rpm for 20 min (4 °C) and allowed to settle at 4 °C for 1 h. The resulting supernatant was carefully collected and filtered through a 0.22 μm membrane. The filtrate was concentrated under reduced pressure using a rotary evaporator until complete ethanol removal was confirmed by the absence of alcohol odor. The concentrated solution was subsequently lyophilized for 35 h to obtain the final freeze-dried KP. The extraction yield was 27.46 g/L.

### 4.3. DSS-Induced UC Model and Drug Intervention

Sixty specific pathogen-free (SPF) BALB/c mice (5–8 weeks old, 18–22 g) were obtained from the Beijing Vital River Laboratory Animal Technology Co., Ltd. (Beijing, China). The mice were housed under controlled environmental conditions (temperature: 20–22 °C; relative humidity: 50–60%; 12/12 h light/dark cycle) with ad libitum access to food and water. After 7 days of acclimatization, the animals were randomly subdivided into 6 groups (n = 10/group): a control group, a DSS model group, a mesalazine treatment group, a low-dose KP treatment (L-KP) group, a medium-dose KP treatment (M-KP) group, and a high-dose KP treatment (H-KP) group. The mice were individually numbered. UC was induced by administering 3% DSS diluted in drinking water ad libitum for 9 consecutive days. From day 3 of DSS administration, therapeutic interventions were initiated: KP-treated groups received daily oral gavage of respective doses (1.35, 2.7, or 5.4 g/kg); mesalazine group received 0.2 g/kg daily oral gavage; control and DSS model groups received equivalent volumes of vehicle. The therapeutic regimens were diluted in drinking water prior to oral gavage. The body weight, stool consistency, and fecal occult blood were monitored daily. Disease activity was quantified using the Disease Activity Index (DAI), calculated as DAI = (weight change score + bloody stool score + loose stool score)/3. Scoring criteria are illustrated in Supplementary Table S1. All procedures were approved by the Animal Welfare Ethics Committee of Beijing Academy of Science and Technology (Approval No. BLARC-SSYY-DW/013-JL/001) on 14 May 2024.

Unless otherwise specified, each group initially contained 10 mice. Different sample sizes were used for individual assays according to assay-specific requirements, tissue availability, and analytical purpose. Accordingly, some analyses, such as serum cytokine measurement and colon length assessment, were performed using all animals in each group, whereas histological and image-based analyses were conducted using representative subsets of samples.

### 4.4. Histopathological Analysis and Serum Cytokine Measurement

Colon tissues were harvested, fixed in 4% paraformaldehyde for 24 h, embedded in paraffin, and sectioned at 5 μm thickness. For histopathological evaluation, representative samples from each group (n = 3/group) were used for H&E staining. Histopathological changes were evaluated using light microscopy (Nikon Eclipse E100, Nikon Corporation, Tokio, Japan) at 200× magnification. For image-based quantification of inflammatory cell infiltration, representative microscopic fields from five samples per group were analyzed (n = 5/group).

Blood samples (n = 10/group) were collected from the orbital venous plexus using heparinized capillary tubes and were centrifuged (4000× *g*, 4 °C, 10 min). The serum supernatant was aliquoted and stored at −80 °C until analysis. Inflammatory cytokines (TNF-α, IL-4, IL-6, IL-10) were quantified using commercial ELISA kits (Uping Bio, Hangzhou, China) according to the manufacturer’s protocol. Optical density was measured at 450 nm using a microplate reader (BioTek Synergy H1, Winooski, VT, USA).

Histological and image-based analyses were performed using representative subsets because these assays were intended to evaluate tissue morphology and local pathological changes, whereas cytokine measurements were carried out using all animals in each group to better reflect systemic inflammatory status.

### 4.5. Preparation of Proteome

Total protein was extracted from the colon tissue of each group (n = 10/group) using the AllPrep DNA/RNA/Protein Mini Kit (QIAGEN, Cat. No. 80004, Hilden, Germany) according to the manufacturer’s instructions. Peptides were prepared using a modified filter-aided sample preparation method as follows: 250 µg of total protein, 100 µL of 7 M guanidine hydrochloride (Sigma-Aldrich, G3272, St. Louis, MI, USA), and 4 µL of 1 M dithiothreitol (DTT, OriLeaf, Lot. S11080, Shanghai, China) were added and the solution was incubated at 55 °C for 1 h. Then, 10 µL of 1 M iodoacetamide (Sigma-Aldrich, I1149, St. Louis, MO, USA) was added and centrifuged at low speed after shaking and mixing. It was left for 30 min under light protection and room temperature, and then centrifuged again. Finally, the filtrate was washed twice with ammonium bicarbonate solution (RHAWN, Lot. 151247, Shanghai, China) and was collected for storage [47]. Then, 25 µL of 0.2 µg/µL trypsin (Thermo Fisher Scientific, 90305, Waltham, MA, USA) was added, and centrifuged at low speed after shaking and mixing. The reaction was left at a constant temperature of 37 °C overnight, was collected by centrifugation and the reaction was terminated by the addition of 2 µL of formic acid (Thermo Fisher Scientific, A117-50, Waltham, MA, USA).

### 4.6. LC-MS Analysis

A high-resolution tandem mass spectrometer Triple TOF 6600+ (SCIEX, Redwood City, CA, USA) connected with an Evosep One LC system was used for peptide detection in both positive and negative ion modes. The Evosep was connected to an analytical column (PepSep, Marslev, Denmark, 15 cm × 150 μm, 1.9 μm beads) at a flow rate of 500 µL/min. The curtain gas was maintained 20 psi, ion source gas 1 and 2 were 20 psi and 0 psi, respectively, using an interface heater temperature of 175 °C, and the ion source temperature at 0 °C. The ion source voltage was controlled at 3500 V. In IDA mode, the mass spectrometry data was acquired, and the TOF mass range was set from 60 to 1200 m/z. The survey scans were acquired in 350 ms and as many as 12 product ion scans were collected a threshold of 60 counts/s and with a 1 + charge-state. Total cycle time was fixed to 1 s. ADC detector equipped with four-anode/channel detection was used. Dynamic exclusion was set for 4 s.

Raw spectral files (.wiff2) were converted to peak lists (.mgf) using Proteome Discoverer 2.1 (Thermo Fisher Scientific) with centroiding enabled and default noise thresholds. For protein identification and quantification, files were analyzed with DIA-NN (v1.9) under the following protocol: a species-specific library was generated [*DDA runs of pooled samples*] and searched against the UniProt Knowledgebase (UniProtKB), specifically the mouse (Mus musculus) proteome entries (Release 2024) at 1% FDR. During acquisition, the mass accuracy was calibrated every 10 samples. Furthermore, in order to evaluate the stability of the LC-MS during the entire acquisition, a pool of 10 samples, referred to as a quality control (10 μL), was acquired for each sample. We identified the depletion step using thermos High Select Top14 abundant protein depletion Mini Spin columns (Thermo Fisher Scientific, A36372).

### 4.7. Molecular Docking Analysis

The three-dimensional (3D) structures of the target proteins were retrieved from the Protein Data Bank database (http://www.rcsb.org, accessed on 21 April 2026) as docking acceptors. The 3D structures of potential activity composition of koumiss in mol2 format were downloaded from the TCMSP database (https://www.tcmsp-e.com/load_intro.php?id=43) as docking ligands. Then, docking was done using AutoDock Vina (v 1.5.2) software. The original ligand of the target proteins active center was removed using Pymol3.1 software, and the target protein was hydrogenated. Binding energy <0 indicated that the compound and protein could spontaneously bind and interact with each other. The lower the energy, the more stable the molecular conformation. Generally, a binding energy of ≤−5.0 kcal/mol indicates a good binding effect. PyMol (v 4.6.0) software was used for visualization.

### 4.8. Immunofluorescence Staining

Paraffin-embedded colon tissue sections (5 μm) were dried at 37 °C for 2 h, deparaffinized in xylene, and rehydrated through a graded ethanol series. Antigen retrieval was performed in 1× Tris-EDTA buffer (pH 9.0; SEVEN, SI120-02, Beijing, China) at 95 °C for 40 min. After cooling to room temperature, endogenous peroxidase activity was blocked with hydrogen peroxide solution (SEVEN, SII25-02, Beijing, China) for 10–15 min. The sections were then washed with TBST (Servicebio, G0004-1L, Wuhan, China) and blocked with 10% goat serum in PBS (Solarbio, SL038, Beijing, China) for 20 min at room temperature.

The sections were incubated overnight at 4 °C with primary antibodies against PPARA, Plin4, and Sorbs1 in a humidified chamber. After washing with TBST, HRP-conjugated secondary antibodies (Proteintech, RGAR011 and RGAM011, Wuhan, China) were added and incubated for 20 min at room temperature. Fluorescence signal amplification was performed using a TSA kit (absin, abs0088, Shanghai, China) according to the manufacturer’s instructions. Briefly, the 520 fluorophore was diluted 1:100 in amplification buffer and applied to the sections for 10 min in the dark at room temperature. Nuclei were counterstained with DAPI (Servicebio, G1012, Wuhan, China) for 10 min. After mounting with antifade medium, images were acquired using a Nikon C2 inverted confocal microscope (Nikon Corporation, Tokyo, Japan). Fluorescence intensity was quantified using ImageJ software (Version 1.54s 26 March 2026).

### 4.9. Statistical Analysis

All statistical analyses were performed using GraphPad Prism (version 8.0, GraphPad Software, San Diego, CA, USA). Data are presented as mean ± standard error of the mean (SEM). Normality of data distribution was assessed prior to parametric analysis. For comparisons among multiple groups, one-way analysis of variance (ANOVA) was conducted, followed by Tukey’s multiple comparison test when equal variance was assumed. When appropriate, Dunnett’s test was applied for comparisons against the model group. A two-sided *p* value < 0.05 was considered statistically significant.

Proteomic data were analyzed using R statistical software (version 4.4.2). Differentially expressed proteins (DEPs) were identified using the DESeq2 package, with thresholds set at |fold change| > 1.5 and false discovery rate (FDR)-adjusted *p* < 0.05. Functional enrichment analysis, including Gene Ontology (GO) and Kyoto Encyclopedia of Genes and Genomes (KEGG) pathway analysis, was performed using the clusterProfiler package. Statistical significance for enrichment analyses was defined as adjusted *p* < 0.05.

## 5. Conclusions

In summary, this study employed an integrated multi-omics framework to elucidate the therapeutic mechanism of koumiss powder (KP) in experimental ulcerative colitis. Network pharmacology predicted lipid-mediated regulation of the PPAR signaling pathway. In vivo validation confirmed that H-KP alleviated disease severity and inflammatory responses. Proteomic analyses demonstrated activation of PPAR-associated effectors, including Plin4 and Sorbs1, which were further validated by immunofluorescence staining in colonic tissues, while molecular docking supported stable interactions between key lipid metabolites—particularly 13(S)-HOTrE and stearoyl ethanolamide—and core regulatory proteins. Collectively, these findings indicate that koumiss exerts protective effects against UC through modulation of PPAR-dependent lipid metabolic and anti-inflammatory pathways. This work not only provides mechanistic support for the ethnomedicinal use of koumiss but also highlights fermented dairy-derived lipid mediators as potential candidates for developing novel dietary or adjunct therapeutic strategies for inflammatory bowel disease.

## Figures and Tables

**Table 1:** Active ingredients of Koumiss.

No.	Name	Formula	Calc. MW
1	13(S)-HOTrE	C_18_H_30_O_3_	294.4
2	16-Hydroxyhexadecanoic acid	C_16_H_32_O_3_	272.4
3	Acetylcholine	C_7_H_16_NO_2_^+^	146.2
4	Arachidonic acid	C_20_H_32_O_2_	304.5
5	Hexadecanamide	C_16_H_33_NO	255.4
6	Lauric acid	C_12_H_24_O_2_	200.3
7	Palmitic acid	C_16_H_32_O_2_	256.4
8	Palmitoleic acid	C_16_H_30_O_2_	254.4
9	Palmitoyl ethanolamide	C_18_H_37_NO_2_	299.5
10	Progesterone	C_21_H_30_O_2_	314.5
11	Stearoyl ethanolamide	C_20_H_41_NO_2_	327.5
12	Tyramine	C_8_H_11_NO	137.2

**Table 2:** Binding energy table of compounds in koumiss docked with PPARA and PPARG.

Active Ingredients	PPARA (kcal/mol)	PPARG (kcal/mol)
Palmitoleic acid	−6.2	−6.3
Arachidonic acid	−7.4	NA
Lauric acid	−5.8	−5.5
Palmitic acid	−5.9	−6.1
16-Hydroxyhexadecanoic acid	NA	−9.3
Progesterone	−6.7	NA
Hexadecanamide	−6.0	−6.0
Stearoyl ethanolamide	−9.5	−10.2
Palmitoyl ethanolamide	−10.5	NA
Acetylcholine	−4.5	−4.5
13(S)-HOTrE	NA	−10.7
Tyramine	−5.7	−5.5

## Data Availability

The original contributions presented in this study are included in the article/Supplementary Material. Further inquiries can be directed to the corresponding authors.

## Supplementary Materials

The following supporting information can be downloaded at: https://www.mdpi.com/article/10.3390/ijms27093821/s1.
