# Supplementary material for: Integrated Multi-Omics Analysis Reveals Activation of the PPAR Signaling Pathway by Koumiss in Experimental Ulcerative Colitis
Source: Int J Mol Sci. 2026 Apr 25;27(9):3821. doi: 10.3390/ijms27093821 (PMC13163921; doi:10.3390/ijms27093821)
Supplement: Supplementary file 1 [file ijms-27-03821-s001.zip › ijms-4213987-supplementary.pdf]

Supplementary Table S1 The scoring system of clinical score

Score	Body weight decrease rate	Fecal property	Hematochezia status
0	0%	Normal	Normal
1	1-5%	Semi loose(+)	Feces with occult blood(+)
2	6-10%	Semi loose(++)	Feces with occult blood(++)
3	11-15%	Loose(+)	Bloody feces(+)
4	> 15%	Loose(++)	Bloody feces(++)

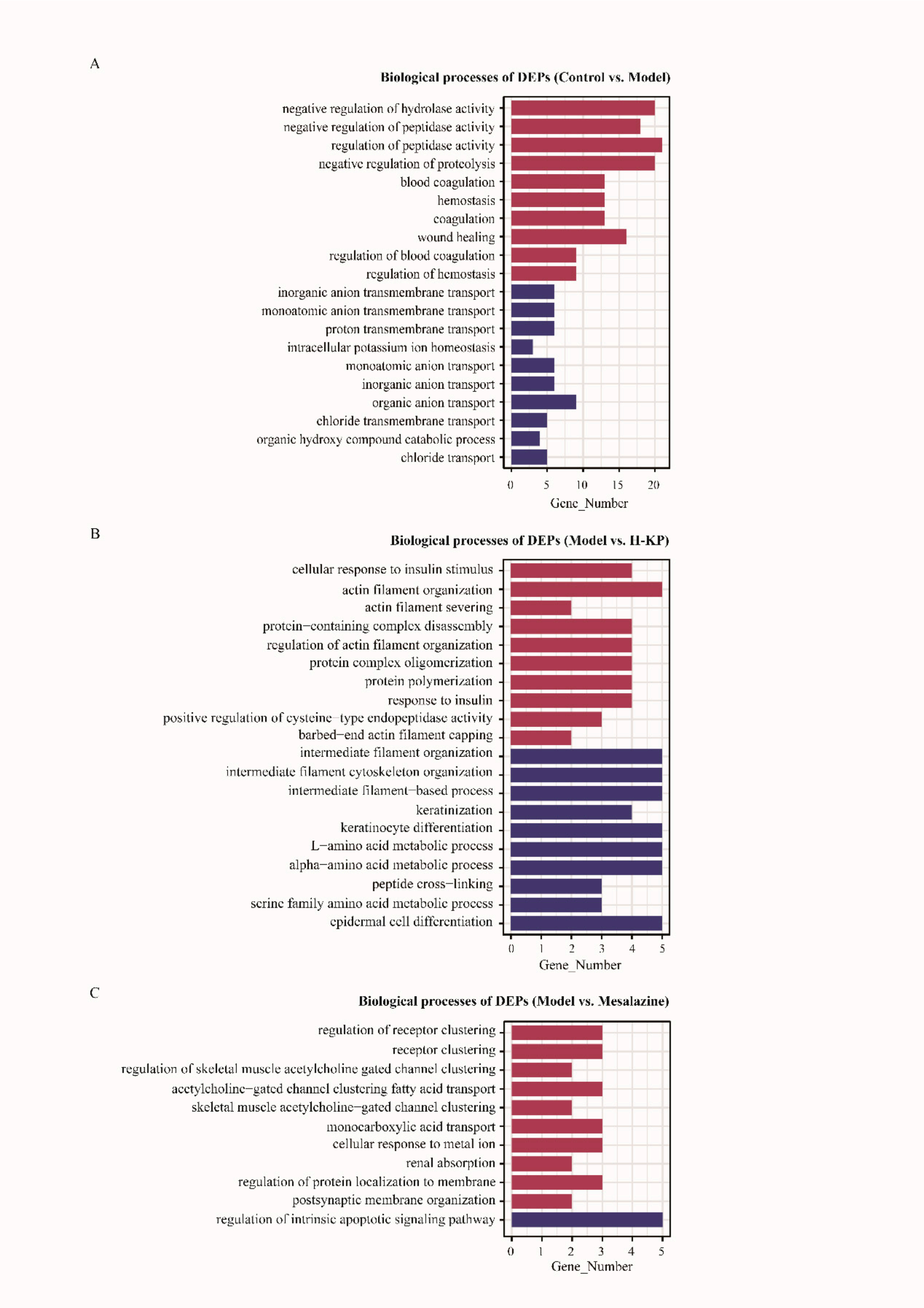

Supplementary Figure S1. Biological process enrichment analysis based on DEPs. Biological process of DEPs in each comparison: (A) model group vs. control group, (B) H-KP vs. model group and (C) mesalazine group vs. model group. Red represents enriched biological process of up-regulated proteins, while blue indicates down-regulated proteins.

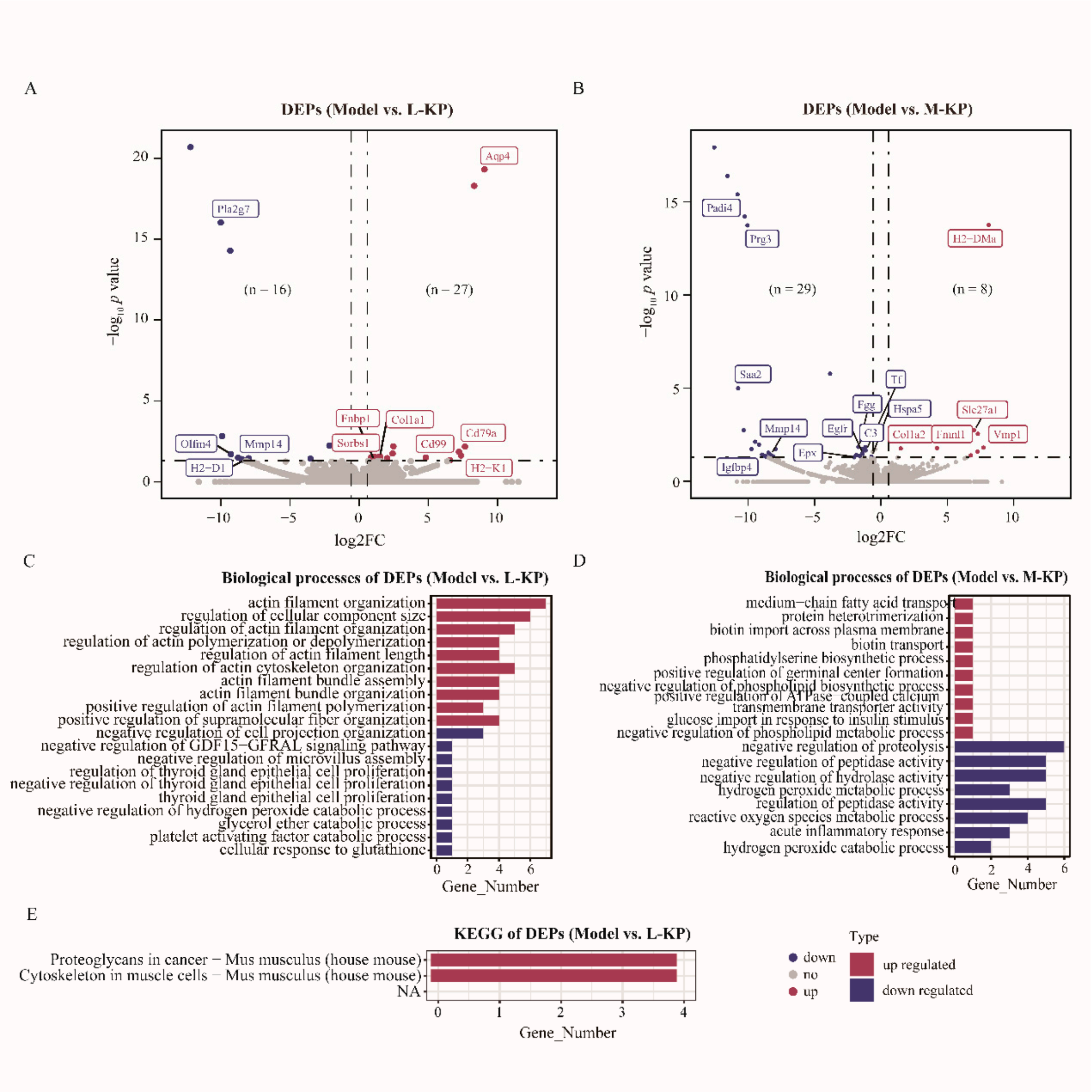

Supplementary Figure S2. Comparative proteomic analysis of DEPs in L-KP and M-KP compared to model group. The volcano plots display up-regulated and down-regulated DEPs (|FC| > 1.5, $p < 0.05$) in L-KP (A) and MKP groups (B) compared to the model group. Biological processes for DEPs in L-KP (C) and M-KP groups (D). (E) KEGG pathway enrichment analysis based on DEPs in L-KP compared to the model group. The red represents up-regulated proteins, the blue represents down-regulated proteins in each comparison.
